# Cost-Effective and High-Throughput LPS Detection via Microdroplet Technology in Biopharmaceuticals

**DOI:** 10.3390/bios15100649

**Published:** 2025-09-30

**Authors:** Adriano Colombelli, Daniela Lospinoso, Valentina Arima, Vita Guarino, Alessandra Zizzari, Monica Bianco, Elisabetta Perrone, Luigi Carbone, Roberto Rella, Maria Grazia Manera

**Affiliations:** 1 CNR NANOTEC–Institute of Nanotechnology, Campus Ecotekne, Via Monteroni, 73100 Lecce, Italy; adriano.colombelli@cnr.it (A.C.); valentina.arima@cnr.it (V.A.); vita.guarino@unisalento.it (V.G.); luigi.carbone@cnr.it (L.C.); 2 CNR IMM–Institute for Microelectronics and Microsystems, Campus Ecotekne, Via Monteroni, 73100 Lecce, Italy; roberto.rella@cnr.it

**Keywords:** microdroplet generation, microfluidics, biosensing, colorimetric sensors, LAL test

## Abstract

Lipopolysaccharides (LPS) from Gram-negative bacteria represent a significant challenge across various industries due to their prevalence and pathogenicity and the limitations of existing detection methods. Traditional approaches, such as the rabbit pyrogen test (RPT) and the Limulus Amebocyte Lysate (LAL) assay, have served as gold standards for endotoxin detection. However, these methods are constrained by high costs, lengthy processing times, environmental concerns, and the need for significant reagent volumes, which limit their scalability and application in resource-limited settings. In this study, we introduce an innovative microfluidic platform that integrates the LAL assay within microdroplets, addressing the critical limitations of traditional techniques. By leveraging the precise fluid control and reaction isolation offered by microdroplet technology, the system reduces reagent consumption, enhances sensitivity, and enables high-throughput analysis. Calibration tests were performed to validate the platform’s ability to detect LPS, using colorimetric measurements. Results demonstrated comparable or improved performance relative to traditional systems, achieving lower detection limits and greater accuracy. This work demonstrates a proof-of-concept miniaturisation of the pharmacopoeial LAL assay. The method yielded low intra-assay variability (σ ≈ 0.002 OD; CV ≈ 0.9% over n = 50 droplets per point) and a LOD estimated from calibration statistics after path-length normalisation. Broader adoption will require additional comparative validation and standardisation. This scalable, cost-effective, and environmentally sustainable approach offers a practical solution for endotoxin detection in clinical diagnostics, biopharmaceutical production, and environmental monitoring. The proposed technology paves the way for advanced LPS detection methods that meet stringent safety standards while improving efficiency, affordability, and adaptability for diverse applications.

## 1. Introduction

Lipopolysaccharides (LPS) from Gram-negative bacteria are significant contaminants in pharmaceutical products, formulation components, and medical devices [1,2]. Known as pyrogens, LPS can induce fever and systemic inflammatory responses when introduced into the human body [3,4]. Due to their prevalence and pathogenicity, LPS are critical biomarkers of biological contamination, widely used to monitor environments and processes susceptible to bacterial contamination, such as water purification systems [5,6]. The biopharmaceutical industry faces the greatest challenge from LPS contamination, as recombinant proteins and therapeutic drugs produced with living organisms, including Gram-negative bacteria, are particularly vulnerable. Sources of contamination include water, excipients, and raw materials used in manufacturing, making continuous monitoring of endotoxins in process streams essential to maintaining safety standards, improving product quality, and optimizing production costs [1,7,8,9,10]. Beyond pharmaceuticals, LPS contamination also affects other sectors, such as food, cosmetics, and textiles, especially those involving engineered nanoparticles [11,12]. In clinical laboratories, LPS can be found in chemicals, glassware, and medical devices designed for injection or implantation. While sterilization effectively eliminates most microbial contaminants, it is ineffective against LPS, underscoring the need for timely and accurate endotoxin screening to ensure patient safety.

The rabbit pyrogen test (RPT) and the Limulus amoebocyte lysate (LAL) assay were the first approved methods for assessing the biological activity of lipopolysaccharides (LPS). The RPT detects pyrogens by measuring the temperature increase in rabbits after intravenous injection of the test solution [13]. While capable of identifying all types of pyrogens, it is now primarily used to validate LAL results due to its drawbacks, including low sensitivity, high costs, lengthy processing times, and ethical concerns related to animal use. In contrast, the LAL assay, introduced by Levin and Bang in 1964, is specifically designed for LPS detection [14]. It leverages the coagulation properties of amoebocyte lysates from the horseshoe crab (*Limulus polyphemus*), where LPS triggers Factor C activation, initiating a coagulation cascade to combat infection. The LAL assay offers accurate quantitative detection of endotoxins and remains the gold standard for LPS measurement despite its limitations.

The limitations of traditional methods, including high reagent consumption, lengthy processing times, and the environmental impact of chemical waste, underscore the need for innovative solutions that can overcome these challenges.

Traditional LAL assays provide accurate quantitative endotoxin detection, with performance depending on the readout format. In gel-clot assays, typical sensitivities fall in the 0.015–0.25 EU mL^−1^ range and processing time can extend up to about two hours, whereas kinetic chromogenic formats are the most sensitive, with reported detection limits down to 0.0002 EU mL^−1^ and assay times around thirty minutes [15,16]. Kinetic turbidimetric assays generally operate quantitatively over 0.001–10 EU mL^−1^ and, depending on the platform (tube versus microplate), require on the order of 50–100 µL of sample; interlaboratory validations report relative accuracy within approximately ±10–20% and coefficients of variation compatible with routine QC [17,18]. Conventional workflows consume tens to hundreds of microlitres of reagents per test and generate millilitre-scale liquid waste (proteins and buffers) requiring controlled disposal; in contrast, microdroplet formats can reduce reagent consumption and associated chemical waste by orders of magnitude while preserving compatibility with optical readouts [15,16].

Recent advancements in the detection of lipopolysaccharides (LPS) have significantly leveraged microfluidic technologies to enhance sensitivity, specificity, and efficiency. For instance, a nanoplasmonic optical biosensing approach integrated into a lab-on-a-chip (LOC) platform has been developed to detect LPS with a detection limit as low as 5 ng/mL, utilizing optical features of nanoplasmonic transducers to create a compact and sensitive device for pharmaceutical quality control [19]. Another study utilized fiber optic probe technology combined with biomimetic self-assembled layers and microfluidics to enhance LPS detection, achieving a lower detection limit of 0.4 ng/mL with high specificity [20]. An innovative micromagnetophoretic cleansing method for LPS removal from biofluids has been proposed, utilizing microfluidics to improve the efficiency of the separation process [21]. A centrifugal microfluidic system has been developed to automate and parallelize the Limulus amebocyte lysate (LAL) assay, reducing reagent consumption by over 90% and ensuring precise and accurate endotoxin detection [22]. The use of surface-enhanced Raman scattering (SERS) integrated with microfluidic arrays enabled the differentiation of endotoxins from various bacterial pathogens at clinically relevant concentrations [23]. A fluorescence turn-on sensing strategy using poly-l-lysine-functionalized carbon dots and microfluidics achieved ultrasensitive detection of LPS, with a detection limit of 68.3 fM [24]. Lastly, a robust off-on fluorescent biosensor based on aggregation-induced emission, combined with microfluidic integration, provided rapid detection and clearance of LPS with a detection limit of 6.97 nM [25]. These studies collectively demonstrate the critical role of microfluidic technologies in advancing the detection capabilities of LPS, offering high sensitivity, specificity, and efficiency across various applications.

An additional advancement in traditional microfluidic technologies is represented by microdroplet microfluidics. This cutting-edge approach builds upon the foundational principles of microfluidics but introduces significant enhancements through the manipulation of discrete droplets, each acting as an isolated microreactor. Unlike traditional continuous-flow microfluidics, where fluids move in a continuous stream, microdroplet microfluidics allows for precise control and individual handling of each droplet. This enhances reaction efficiency and minimizes cross-contamination, making it particularly suitable for high-throughput screening and complex biochemical assays.

The coupling of microdroplet microfluidics with optical investigation methods further enhances its utility, particularly in the detection of bacterial endotoxins. By integrating optical devices such as microscopes, CMOS cameras, and photodiodes with microfluidic chips, it is possible to conduct real-time monitoring and analysis of the reactions occurring within the droplets. This optical detection can measure absorbance, fluorescence, and other optical properties, providing a rapid and sensitive means to detect endotoxins like lipopolysaccharides (LPS) [26,27].

In this study, a microfluidic platform for LPS detection was developed by integrating the Limulus Amebocyte Lysate (LAL) assay within microdroplets. The setup incorporates a visual investigation system, including an inverted microscope and CMOS camera, alongside an optical characterization system for absorbance and transmittance measurements. Calibration tests were conducted in two phases to ensure the system’s accuracy. Colorimetric calibration was achieved by analysing the optical properties of droplets containing varying dye concentrations, enabling precise absorbance measurements at specific wavelengths. Traditional LAL assays typically use 100 µL of reagent, which is costly and generates significant chemical waste. This work progressively minimized the reagent volume to 5 µL and further minimizes by pushing up to the volume contained within a single microdroplet. This innovative approach addresses key challenges in endotoxin detection, including cost, waste, and scalability, offering a practical and environmentally friendly alternative to traditional methods. Lower reagent volumes directly lead to significant cost savings, particularly in large-scale or industrial testing, while also reducing chemical waste and enhancing the environmental sustainability of the process. Despite the reduction in reagent volume, the assay maintained its reliability in detecting endotoxins and exhibited enhanced sensitivity, enabling the detection of lower endotoxin concentrations with greater accuracy.

## 2. Experimental Section

### 2.1. Droplet Generation & Control

**Figure 1:**
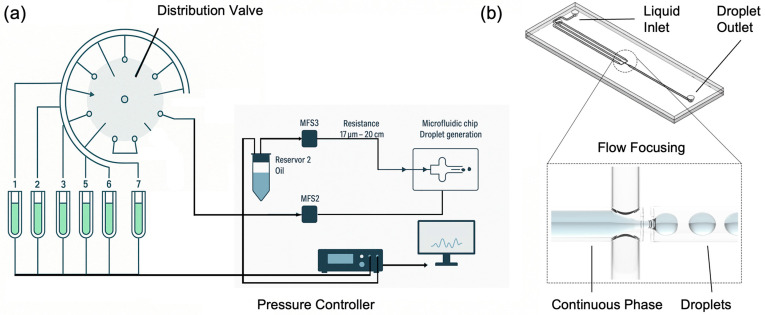
(**a**) Schematic representation of the droplet generation system. The setup includes a distribution valve, multiple pressurized reservoirs, flow rate sensors, an oil reservoir, a microfluidic chip, and a pressure controller. (**b**) Generic design of the flow-focusing microfluidic chip used in this study. This configuration ensures the formation of uniform droplets by controlling the interaction between the dispersed aqueous phase and the continuous oil phase.

The setup for droplet generation is reported in Figure 1a and is composed of a distribution valve (ElveFlow–Microfluidic Distribution Valve, Paris, Île-de-France, Francia), multiple pressurized reservoirs, flow rate sensors (ElveFlow–Microfluidic Flow Sensor–MFS), an oil reservoir, a microfluidic chip, and a pressure controller (ElveFlow–OB1 MK4). This system offers the advantage of high precision and flexibility, allowing for the customizable generation of droplets with various contents. The distribution valve enables easy modification of droplet composition by selecting different fluid inputs, making the system highly versatile for various applications.

The heart of this droplet generation system is the OB1 pressure controller, which ensures precise control over the entire setup. OB1 is connected to the pressurized reservoirs providing real-time adjustments to maintain optimal conditions for droplet formation. It is integrated with a feedback loop system and connected to a computer running ELEVEFLOW software, which offers a user-friendly interface for monitoring and controlling the process. At the core of the fluid management system is the MUX distributor (distribution valve), equipped with eight outputs labelled from 1 to 8. This valve manages the flow from multiple pressurized reservoirs, allowing the selection of different fluid inputs. This configuration enables the generation of droplets with varying compositions, enhancing the system’s adaptability. Flow rate sensors are installed to monitor and control the fluid flow from the distribution valve. These sensors are essential for maintaining the accuracy and consistency of droplet formation. The system also includes an oil reservoir connected via a dedicated flow sensor that regulates the flow of oil used in the droplet generation process. The microfluidic chip, where the actual droplet generation occurs, is connected to the flow system with precise flow resistances. These resistances enable to highly control the flow rates and pressures, ensuring that the fluids enter the chip at the desired rates to form uniform droplets. The microfluidic chip is designed to facilitate high-throughput droplet production, making the system suitable for a variety of research and industrial applications. In this study, the microfluidic chips selected for droplet generation utilized a flow-focusing configuration (Figure 1b). This approach, a common and effective method in microfluidics, involves the co-axial flow of the dispersed aqueous phase and the continuous oil phase through a contraction region. The interaction of these phases generates an elongated filament that breaks into uniform droplets. This configuration allows precise control over droplet volumes by adjusting the flow rate ratios and channel dimensions. The microfluidic chip used in this study is the Micronit Classic Droplet Generator–L, designed for generating highly monodisperse droplets. We utilized the water-in-oil (W/O) configuration, where the chip’s surface is coated for hydrophobic properties, ensuring effective droplet stabilization within an oil-based continuous phase. This version features a large channel configuration, with a nozzle height of 50 µm, a channel width of 500 µm, and a channel height of 100 µm. The chip is fabricated from high-quality borosilicate glass, ensuring chemical inertness and biocompatibility. It incorporates precision-etched microfluidic channels to guarantee reproducible results and a single-etched nozzle for stable droplet generation. This setup is particularly suitable for applications such as digital PCR, single-cell analysis, nanoparticle synthesis, and encapsulation, offering precise control over droplet size and frequency by tuning the flow rates of the dispersed and continuous phases.

### 2.2. Optical Characterization Setup

**Figure 2:**
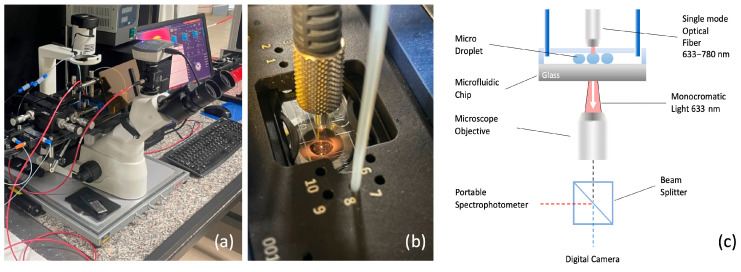
(**a**) Overview of the experimental setup, featuring an inverted fluorescence microscope equipped for simultaneous imaging, transmittance, and absorbance measurements. (**b**) Close-up view of the microfluidic chip positioned over the microscope objective, with a fiber optic probe delivering monochromatic or polychromatic light for optical analysis. (**c**) Schematic representation of the system’s working principle. A single-mode optical fiber directs monochromatic light (633 nm) through the microdroplets in the microfluidic chip. The transmitted and scattered light is collected by the microscope objective and split between a high-resolution digital camera for imaging and a portable spectrophotometer for spectroscopic measurements.

Figure 2a shows the optical characterization setup developed in this study, designed for a comprehensive analysis of the optical properties of microdroplets. The system incorporates an inverted fluorescence microscope (Optika XDS-3FL, Optika S.R.L., Italy, Ponteranica (BG)) capable of performing simultaneous imaging, transmittance, and absorbance measurements. It is designed for both static and dynamic assessments, providing versatility across a wide range of applications. Specifically, the setup was developed to conduct colorimetric analyses on droplets, enabling precise measurements of their optical properties under varying experimental conditions (Figure 2b).

The key components include a high-sensitivity camera for imaging (CMEX DC 5000, Euromex Microscopen bv, VB Duiven, The Netherlands), a portable spectrometer for transmittance and absorbance analysis (Avantes AVS-MC2000-5, Avantes B.V., NS Apeldoorn, The Netherlands), wavelength ranging between 300–1100 nm), and a light source that can be configured as either monochromatic or polychromatic, depending on the type of absorbance measurement required. The schematic in Figure 2c illustrates the integration of these components, ensuring precise control and synchronization between the different measurement modes.

A light source, depicted as a red beam, is directed downward through the droplet towards a microscope objective positioned below the glass slide of the microfluidic chip. Colorimetric measurements, involving droplets with varying concentrations of dye, employ a polychromatic light source (deuterium halogen light source). The broader spectrum enables detection of absorbance changes across multiple wavelengths, which corresponds to the dye’s characteristic absorption profile. This allows for a more accurate determination of concentration by analyzing the absorbance peaks at specific wavelengths.

The microscope objective, located directly beneath the microfluidic chip, performs both imaging and absorbance measurements simultaneously, facilitated by a beam splitter. The imaging signal is routed to a high-resolution camera for detailed visualization of the droplets, while the transmitted or emitted radiation from the droplets is directed to a portable spectrophotometer for optical analysis. This dual functionality enables the concurrent collection of visual and spectroscopic data, providing a comprehensive understanding of the optical properties of the droplets.

### 2.3. Experimental Set-Up for Colorimetric Test

For calibration of the optical detection system, we used aqueous methylene blue (MB) solutions under polychromatic halogen illumination. A saturated MB stock solution was first prepared at 25 °C (excess solid added, equilibrated, and filtered). The maximum solubility at this temperature is about 43.6 g L^−1^ (≈4.36% *w*/*v*). The calibration standards reported in the manuscript as “0%, 5%, 15%, 30%, 45%, and 60% MB solutions” actually refer to volumetric dilutions of the saturated stock solution with deionised water (i.e., % *v*/*v* of the stock, not % *w*/*v* of MB). The corresponding approximate mass concentrations were: 5% *v*/*v* ≈ 2.2 g L^−1^, 15% *v*/*v* ≈ 6.5 g L^−1^, 30% *v*/*v* ≈ 13.1 g L^−1^, 45% *v*/*v* ≈ 19.6 g L^−1^, 60% *v*/*v* ≈ 26.2 g L^−1^. All concentrations were therefore well below the solubility limit of MB in water at 25 °C. Methylene blue was employed only for calibration of the optical system and was not part of the LAL assay.

This approach was designed to simulate the presence of chromogenic elements within the droplets, such as those used in Limulus Amebocyte Lysate (LAL) tests. By calibrating the system with these known dye concentrations, it is possible to establish a baseline optical response, ensuring accurate measurement of the optical properties of droplets during subsequent LAL tests.

The advantage of this calibration lies in its ability to precisely tune the optical detection system before analyzing more complex samples, such as those in LAL tests where the presence of endotoxins leads to a chromogenic reaction. This preliminary calibration allows for more accurate differentiation between droplets based on their absorbance characteristics, leading to improved sensitivity and specificity during the actual analysis.

In this experiment, a polychromatic halogen light source was used, with emission covering the spectral range of interest. This choice enables the investigation of specific absorption bands within the substances present in the droplets. The use of a broad-spectrum light source allows for the detection of characteristic absorbance peaks and variations across a range of wavelengths, providing a comprehensive analysis of the optical properties of each droplet. As a result, both static and dynamic measurements of droplet absorbance can be captured.

By integrating the absorbance curve over a defined spectral range, it is possible to track the dynamics of each droplet’s passage through the detection zone, thereby distinguishing subtle changes in their optical properties. This method enables a real-time, automated analysis of hundreds or even thousands of droplets, as variations in absorbance signal can be monitored dynamically over time. Such analysis allows for a detailed statistical study of droplet populations, which highlights how shifts in optical properties can be effectively discriminated through this calibrated approach. This capability is particularly beneficial for applications requiring high-throughput analysis and precise monitoring of sample changes, such as in LAL testing.

### 2.4. Experimental Set-Up for Microdroplet Endotoxin Detection

This experiment involved adapting the Limulus Amebocyte Lysate (LAL) test to a microdroplet format, focusing on progressively reducing the volume of reagents used for the assay. Initially, the test employed standard volumes of 100 µL, as commonly used in commercial LAL test wells. This phase served as benchmark for the transition to smaller volumes. The next phase reduced the volume to 5 µL, which allowed for a preliminary assessment of the test’s performance with a significantly lower quantity of reagents while maintaining the integrity of the reaction. The final stage involved further miniaturization, utilizing the volume of solution contained within a single microdroplet 0.02 µL. This transition required precise calibration of the microfluidic system to ensure that each droplet maintained consistent size and composition.

Endotoxin standard solutions (0–0.5 EU mL^−1^) were prepared by serial dilution of a certified Control Standard Endotoxin (Chromogenic LAL Endotoxin Assay Kit, FUJIFILM Wako Pure Chemical Corporation, Osaka, Japan, Cat. No. 296-83601, 20 tests). The chromogenic Limulus Amebocyte Lysate reagent was reconstituted according to the manufacturer’s instructions. The chromogenic substrate (Boc-Leu-Gly-Arg-pNA) releases p-nitroaniline (pNA), a yellow chromophore typically detected at 405 nm; in our setup, absorbance changes were monitored using a broadband white light source.

Using the three different volumes 100 µL, 5 µL, and 0.02 µL we tested the detection of known endotoxin concentrations of 0.01, 0.025, 0.05, and 0.10 EU/mL. This step was crucial for assessing the sensitivity of the LAL test across varying scales and ensuring that the miniaturized approach could accurately detect low levels of endotoxins. By conducting the assay with these specific concentrations, the study aimed to directly compare the detection performance between the traditional, intermediate, and microdroplet volumes.

The results provided insights into how sensitivity varied with the reduction in reaction volume, allowing for an evaluation of whether the microdroplet-based method could match or even surpass the detection capabilities of larger-volume assays. This comparison helped validate the effectiveness of the miniaturized system for detecting trace levels of endotoxins, which is essential for applications where high sensitivity is required.

To ensure reliable detection within each droplet, the reaction conditions, including temperature and mixing, were carefully controlled throughout the microfluidic channel. For droplet generation, the pre-mixed aqueous solution (endotoxin standard and LAL reagent) was loaded into a pressurized reservoir and introduced into the chip at 50–100 mbar, while the continuous oil phase (HFE-7500 fluorinated oil supplemented with 1% w/w Pico-Surf surfactant) was pressurized at 250–300 mbar. These conditions corresponded to flow rates of ~1 µL/min (aqueous) and ~5 µL/min (oil), producing droplets at ~10 Hz with diameters of 300–350 µm.

These conditions ensured that the LAL reaction progressed uniformly, facilitating accurate endotoxin detection even in the reduced reaction volumes.

The experiment also involved continuous monitoring of the optical signals generated by the reaction within each droplet. Using a portable spectrophotometer aligned with the microfluidic channel, the system captured any changes in optical properties, which corresponded to the presence of increasing endotoxins concentrations in the droplets.

## 3. Results and Discussion

### 3.1. Micro-Droplet Generation and Morphology Characterization

**Figure 3:**
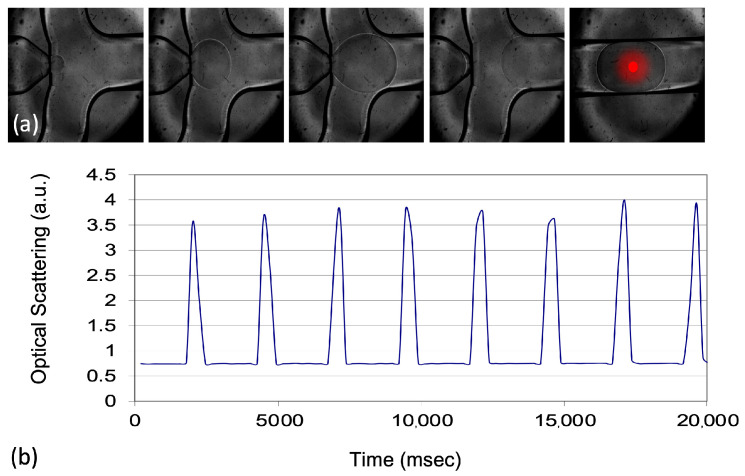
(**a**) Designs and custom-made droplet generation setups. (**b**) Optical scattering acquired as each individual droplet passes through the microfluidic channel.

The validation of the droplet generation and control system was a critical step in ensuring the reliability of the microfluidic approach used in this study. The system was tested using both commercial microfluidic chips of various designs and custom-made droplet generation setups. Different configurations were explored to optimize the droplet formation process, allowing the production of droplets in a range of sizes. This testing phase was essential for determining the most suitable setup for the subsequent experiments. After evaluating various options, a commercial droplet generation system was selected for use in the calibration of the optical system and the miniaturized LAL test. This system was chosen because it allowed to produce droplets with a consistent diameter of 300–350 µm as illustrated in Figure 3.

The droplet diameter was selected to approximately match the microchannel width (~500 µm) so that the probing beam traverses only the aqueous core of each droplet. This geometric matching minimises the portion of the optical path spent in materials with refractive index mismatches (PDMS/glass and fluorinated oil). If droplets are substantially smaller than the channel width, an annular oil layer remains in the beam path and the ray crosses multiple interfaces (PDMS–oil–water–oil–PDMS), amplifying edge-related scattering and refraction artefacts that distort absorbance readouts. Conversely, much larger droplets are harder to generate reproducibly with the commercial chip at the desired throughput. Literature on optical detection in droplet microfluidics reports these edge effects and recommends maximising the effective optical path within the absorbing phase to stabilise the readout [27]. In agreement with the Beer–Lambert law, increasing the optical path length improves absorbance sensitivity; indeed, studies addressing the limited path-length problem use wider gaps (≈500 µm) and larger droplets (≈1.3–1.5 µL) to boost sensitivity when geometry allows (see examples discussed by Pärnamets et al., [27]). Our choice of ~0.02 µL (20 nL) droplets represents a practical compromise between path length (sensitivity), droplet stability, and high-throughput generation with the adopted chip.

### 3.2. Colorimetric Test and Results

**Figure 4:**
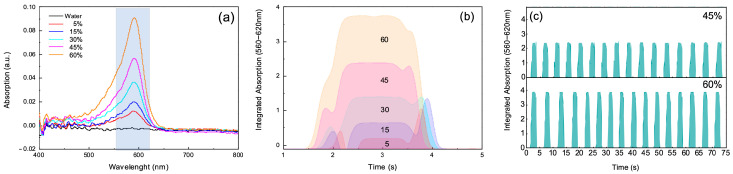
(**a**) Absorbance spectra of microdroplets with dye concentrations of 5%, 15%, 30%, 45%, and 60%, showing increasing peaks with concentration. (**b**) Integrated absorbance values for each concentration, illustrating the system’s ability to distinguish between different dye levels. (**c**) Time-series absorbance data of individual droplets, highlighting consistent droplet generation and stable measurements.

The results of the colorimetric test, as shown in Figure 4, demonstrate the system’s response to varying concentrations of dye within the microdroplets. Figure 4a displays the absorbance curves for droplets containing increasing dye concentrations of 5%, 15%, 30%, 45%, and 60%. The absorbance peaks increase proportionally with the concentration of dye, confirming the ability of the optical detection system to differentiate between different chromogenic contents within the droplets. The absorbance curves clearly show that as the dye concentration increases, the intensity of the peak within the highlighted spectral range becomes more pronounced. This trend is consistent with the Beer-Lambert law, where absorbance is directly related to the concentration of the absorbing species. Figure 4b illustrates the integrated absorbance of one single droplet over a defined spectral range (550–620 nm) for each concentration level, visually represented as a stacked area plot. This representation highlights the distinction between different dye concentrations, showing a progressive increase in absorbance from 5% up to 60%. The integrated absorbance data validates the system’s capacity to quantify dye concentration within droplets, even when small differences in concentration are present.

Furthermore, Figure 4c shows a sequence of time-series data capturing the absorbance changes in individual droplets as they pass through the optical detection area. The consistent shape and size of the peaks indicate the uniformity of droplet generation and the stability of the absorbance measurements across all tested concentrations. The ability to maintain such consistency is crucial for the automated analysis of hundreds or thousands of droplets, allowing for high-throughput, reliable measurements.

These results confirm that the optical detection system, when calibrated with known concentrations, can accurately measure the absorbance properties of microdroplets containing chromogenic substances. The distinction between absorbance levels at each concentration supports the use of this method for precise and real-time monitoring of optical properties in complex samples, such as those encountered in miniaturized LAL tests.

### 3.3. Endotoxin Detection

**Figure 5:**
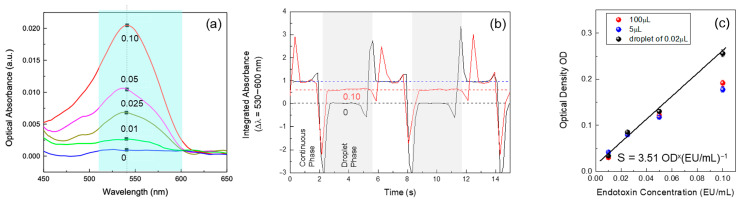
(**a**) Absorbance spectra of microdroplets with increasing endotoxin concentrations (0.01, 0.025, 0.05, 0.10 EU/mL) and microdroplets without endotoxins (0). Higher concentrations show increased absorbance peaks. (**b**) Time-series data of absorbance changes for microdroplets passing through the detection area, with gray areas indicating phases of microdroplet presence. (**c**) Comparison of detection sensitivity across different volumes (100 µL, 5 µL, 0.02 µL and a single microdroplet), showing consistent sensitivity despite reduced reagent volumes.

The results of the miniaturized LAL test, presented in Figure 5, demonstrate the system’s ability to detect endotoxin concentrations across different reaction volumes—100 µL, 5 µL, and 0.02 µL inside a single microdroplet. Figure 5a shows the absorbance spectra of microdroplets containing varying endotoxin concentrations, of 0.01, 0.025, 0.05, and 0.10 EU/mL. The absorbance curves indicate that higher endotoxin concentrations produce stronger absorbance signals, with a notable intensity peak shift observed between the highest and the lower concentrations.

The absorbance differences between these concentrations are crucial for evaluating the sensitivity of the microdroplet-based LAL test. As seen in Figure 5a, the separation between the absorbance curves for different endotoxin levels confirms that the system can reliably distinguish even subtle variations in concentration. The clear distinction between the sample without endotoxin and the lowest detectable concentration (0.01 Eu/mL) further demonstrates the method’s capability to identify trace levels of endotoxins.

Figure 5b provides time-series data of the absorbance changes for microdroplets as they pass through the optical detection area, with the gray area corresponding to individual droplets containing 0.10 EU/mL endotoxin concentration. Droplets without endotoxins and those with the highest endotoxin concentration were compared in this graph. The signal for the highest concentration is consistently higher than those for the lower concentrations, indicating the dynamic response of the system to varying levels of endotoxins. The stable baseline observed for droplets without endotoxins confirms that the system remains unaffected by noise in their absence, underscoring its precision. The spikes observed in the dynamic curve are attributed to the inhomogeneous scattering of light at the circular meniscus of the droplets—an effect that is not present when analyzing the central portion of each droplet, where the optical properties remain more uniform.

Finally, Figure 5c shows a comparison of the detection sensitivity for each volume—100 µL, 5 µL, and 0.02 µL. The graph plots the Optical Density (OD) values against the endotoxin concentrations. To ensure comparability across different sample volumes, OD was calculated using a pathlength correction approach based on the application of the Beer-Lambert law, normalizing the absorbance values to account for variations in the optical pathlength between droplets and volumes of different sizes. Since the pathlength varies with droplet size and shape, a scaling factor was applied based on the known dimensions of each droplet type, ensuring that OD values were directly comparable across all conditions.

To compare absorbance across droplets of different diameters, optical density values were corrected for the effective optical path length according to the Beer–Lambert law:

*A* = *ε*
*c*
*L*_*eff*_ (1)

where A is the measured absorbance (OD), ε the molar extinction coefficient, c the analyte concentration, and L_eff_ the effective path length through the absorbing phase. In our geometry, L_eff_ is approximated by the droplet diameter measured at the interrogation plane (microscopy-calibrated), because the probe beam traverses the aqueous core.

To account for variations in path length, we report a normalised absorbance:

A_norm_ = (A_meas_/L_eff_) × L_ref_ (2)

where A_meas_ is the raw absorbance returned by the spectrometer, L_eff_ is expressed in the same length units as L_ref_, and L_ref_ = 10 mm (conventional cuvette path length). Unless otherwise stated, OD values plotted in the figures correspond to A_norm_.

This normalisation assumes (i) uniform concentration within the droplet at the measurement point and (ii) single-pass absorption with negligible interface artefacts, consistent with our beam crossing the aqueous core. Use of path-length normalisation in microfluidic absorbance readouts is standard practice and aligns with Beer–Lambert-based calibration procedures [27,28].

For each calibration point reported in Figure 5c, the optical density (OD) was calculated as the mean value obtained from 50 individual microdroplets analysed under identical conditions. The standard deviation (σ) was computed from the distribution of these 50 measurements, yielding a typical σ ≈ 0.002 OD. Considering a mean absorbance of ~0.225 OD, this corresponds to a coefficient of variation (CV) of approximately 0.9%. Each concentration point was measured in three independent experiments, and the reported error statistics were consistent across replicates. Unless otherwise stated, error bars in the calibration plots represent these standard deviations.

The results demonstrate that the miniaturized LAL test performed in single microdroplets provides sensitivity s = 3.51 (OD × (EU/mL)^−1^) comparable to that of the larger-volume assays and a LOD of about 0.002 EU/mL. However, the microdroplet approach achieves this sensitivity while using significantly smaller reagent volumes, offering a more efficient and sustainable method for endotoxin detection. Overall, the results validate the use of microdroplet-based LAL testing as a viable alternative to traditional methods, maintaining sensitivity and accuracy even with reduced volumes. This capability is particularly valuable for applications requiring high-throughput screening and precise detection of low endotoxin concentrations, making it suitable for biopharmaceutical testing and other critical applications. Our aim in this study was not to compete with emerging detection modalities such as SERS or fluorescence assays, which indeed can achieve very low detection limits under specific conditions, but rather to demonstrate that a pharmacopoeial LAL workflow can be successfully miniaturised into the nanolitre droplet regime. This choice prioritises compatibility with regulatory practice, established reagents, and acceptance criteria over the development of a new biochemical transduction principle. We therefore did not include a performance benchmarking with SERS or fluorescence assays, as our intention is to preserve the biochemical specificity and regulatory recognition of the LAL test while addressing practical limitations of reagent consumption, waste generation, and assay scalability. Future work will focus on further validation and miniaturisation of the optical readout (fibre-based coupling, compact spectrometers), rather than on replacing the biochemical transduction mechanism. The present study should be considered a proof-of-concept implementation of a microdroplet LAL assay that achieves analytical performance comparable to conventional formats while drastically reducing reaction volume. We do not claim immediate replacement of the gold-standard workflows; rather, we outline a feasible route towards broader adoption. Our bench-top set-up was assembled from commercial modules to accelerate prototyping (pressure controller and compact spectrometer), but the underlying functions—stable water-in-oil droplet generation and single-pass optical readout—are platform-agnostic. In practice, the same functions can be reproduced with syringe pumps or low-cost piezoelectric pump modules for flow control, and LED/photodiode pairs or miniature fibre-coupled spectrometers for detection. Prior work in droplet microfluidics and compact optical readouts supports both modularity and miniaturisation in portable, high-throughput formats [22,26,27]. A plausible engineering path includes (i) replacing the bench-top pressure unit with compact pump drivers; (ii) integrating a flow-focusing droplet generator with 405 nm LED illumination and a silicon photodiode on a small PCB (fibre or free-space coupling); and (iii) packaging with basic temperature control and embedded acquisition/processing. These steps are outside the scope of the present study but are technically compatible with our concept. Finally, we emphasise that formal substitution of pharmacopoeial LAL methods will require head-to-head comparisons under identical conditions, robustness and interference testing across matrices, and alignment with validation criteria.

## 4. Conclusions & Future Perspective

In this study, we present a microdroplet-based system for performing the Limulus Amebocyte Lysate (LAL) test, addressing key challenges in endotoxin detection within biopharmaceutical applications. By miniaturizing the LAL assay into microdroplets, we demonstrate a significant reduction in reagent consumption while maintaining—or even enhancing—the sensitivity of endotoxin detection. Progressively decreasing the reaction volume from 100 µL to just 0.02 µL, we validate the system’s ability to detect low endotoxin concentrations with high precision.

Our results confirm that the miniaturized system preserves detection sensitivity across different endotoxin concentrations (0.01–0.10 EU/mL), achieving performance comparable to or exceeding that of conventional larger-volume assays. This approach offers multiple advantages, including reduced operational costs, lower reagent consumption, and a minimized environmental footprint, making it a scalable and sustainable solution for endotoxin testing in both industrial and clinical settings. Furthermore, the integration of real-time optical detection within the microfluidic platform enables high-throughput testing, a critical factor for improving efficiency in large-scale applications. We have demonstrated a proof-of-concept microdroplet implementation of the LAL assay that maintains comparable analytical performance to conventional formats while reducing reaction volume by orders of magnitude. The architecture is modular and platform-agnostic, enabling migration from bench-top controllers to compact pump modules and LED/photodiode detection, which suggests a credible route towards portable devices for high-throughput endotoxin screening. Nevertheless, broad adoption will depend on further comparative validation against pharmacopoeial methods, extended robustness and interference studies in relevant matrices, and standardisation of operating procedures and acceptance criteria. These activities, while beyond the present scope, are essential to support translation into pharma and clinical settings.

The next phase of development will focus on further reducing droplet volumes to just a few picoliters, maximizing reagent efficiency while preserving detection sensitivity and accuracy. From a socioeconomic perspective, the scalability of this technique has the potential to lower costs associated with drug development and manufacturing, thereby making safe and effective therapies more accessible. Additionally, by minimizing reagent usage, this method could contribute to the conservation of horseshoe crabs, which play a vital role in the production of traditional LAL reagents.

Looking ahead, the versatility of this microdroplet platform opens new avenues for research and development. Future efforts could focus on integrating the system with portable and point-of-care devices, enabling rapid endotoxin testing in decentralized settings such as clinics or field diagnostics. Finally, by leveraging advances in automation and data analysis, this microdroplet platform could be incorporated into fully automated systems, enabling real-time monitoring and quality control in pharmaceutical manufacturing, ultimately enhancing both efficiency and safety in production processes.

## Data Availability

The original contributions presented in this study are included in the article. Further inquiries can be directed to the corresponding authors.
